# Supplementary material for: New, simplified versus standard photodynamic therapy (PDT) regimen for superficial and nodular basal cell carcinoma (BCC): A single-blind, non-inferiority, randomised controlled multicentre study
Source: PLoS One. 2024 Mar 8;19(3):e0299718. doi: 10.1371/journal.pone.0299718 (PMC10923430; doi:10.1371/journal.pone.0299718)
Supplement: S1 File — (DOCX) [file pone.0299718.s003.docx]

**Author summary**

**Why was this study done?**

Basal cell carcinoma (BCC) is the most common type of skin cancer in the adult white population, with the highest occurrence in Australia, with over 1000 cases per 100000 individuals annually. Photodynamic therapy (PDT) is a treatment method that involves the use of a light-sensitive medicine in a cream or gel formulation and a light source to kill cancer cells while providing a good cosmetic result. The method has been used for over 20 years to treat BCC. The standard regimen consists of two treatment sessions, established without prior randomized controlled studies. Simplifying this treatment could reduce costs, be easier to organize, and cause less discomfort for patients.

**What did the researchers do and find?**

We conducted a single-blind, non-inferior, randomised, controlled study across seven centers in Norway and aimed to investigate if a simpler and more flexible PDT regimen could replace the use of the standard two sessions approach for selected BCC.

Together 402 BCC were randomly assigned to receive either a single PDT with the possibility of re-treatment for non-responsive tumours or the standard two-session treatment. Medical doctors, unaware of the treatment allocation, regularly assessed the treated areas for treatment effectiveness and cosmetic outcomes. The final assessment occurred 36 months after PDT, revealing that the simplified PDT regimen was 11.6% less effective than the standard double treatment, with no apparent cosmetic advantages.

**What do these findings mean?**

This study provides robust evidence regarding the choice between one or two PDT sessions for treating BCC. The findings endorse the current practice of administering two PDT sessions with a one-week interval, providing valuable guidance for clinical practice.
