# Supplementary material for: New, simplified versus standard photodynamic therapy (PDT) regimen for superficial and nodular basal cell carcinoma (BCC): A single-blind, non-inferiority, randomised controlled multicentre study
Source: PLoS One. 2024 Mar 8;19(3):e0299718. doi: 10.1371/journal.pone.0299718 (PMC10923430; doi:10.1371/journal.pone.0299718)
Supplement: S2 File — (PDF) [file pone.0299718.s004.pdf]

# CLINICAL TRIAL PROTOCOL

| | |
|---|---|
| **Study title** | **A randomized, controlled-blinded, multi-centre study of photodynamic therapy with methyl-aminolevulinate comparing a simplified regime with the approved regime in patients with clinical low-risk superficial and nodular basal cell carcinoma.** |
| **Investigational product:** | **Methyl-aminolevulinate** |
| **Study code:** | **NTNU 2011-01** |
| **EudraCT number:** | **2011-004797-28 NO 20120327** |
| **Protocol version:** | **Final version revised, 2012-03-27** |

**SPONSOR INVESTIGATOR**
Prof. Magne Børset
Department of Cancer Research and Molecular Medicine, Faculty of Medicine, Norwegian University of Science and Technology (NTNU), Trondheim, Norway. Trondheim

**RESPONSIBLE LOCAL INVESTIGATOR**
Prof. Cato Mørk
Department of Cancer Research and Molecular Medicine, Faculty of Medicine, Norwegian University of Science and Technology (NTNU), Trondheim, Norway. Trondheim

Responsible for research at the various hospitals/ contact persons: St. Olav's University Hospital; Vigleik Jessen, Haukeland University Hospital; Lisbeth Rustad, Stavanger University Hospital; Stein T. Nilsen, Oslo University Hospital, Rikshospitalet; Per M. Sandset, Oslo University Hospital, Radiumhospitalet; Kristin Bjordal, Førde District General Hospital; Øystein Vatne

**RESPONSIBLE COORDINATING INVESTIGATOR**
Name: Dr. Eidi Christensen
Department of Cancer Research and Molecular Medicine, Faculty of Medicine, Norwegian University of Science and Technology (NTNU), Dept. of Dermatology, St. Olav's Hospital HF Trondheim, Norway. Trondheim

**BIOSTATISTICIAN**
Prof. Eirik Skogvoll, Unit for applied clinical research, Faculty of Medicine, Norwegian University of Science and Technology (NTNU), Trondheim, Norway

**OTHER PARTICIPATING CENTERS**
C Mørk, MD, Ph.D. Institute of Cancer Research and Molecular Medicine, Faculty of Medicine, Norwegian University of Science and Technology (NTNU) Trondheim, Norway and Akershus Dermatological Centre, Skårersletta 18, 1473 Lørenskog.

S Kroon, MD. Department of Dermato-Venereology, Stavanger University Hospital, Division of surgery, Helse Stavanger HF, Stavanger, Norway.
T Warloe, MD, Ph.D. Department of Surgery, Oslo University Hospital Rikshospitalet, Oslo, Norway.
P Helsing, MD. Department of Dermatology, Oslo University Hospital, Rikshospitalet, Oslo, Norway.
AM Soler, MD, PhD, Hudlegene på Holtet DA, Kongsveien 94, 1177 Oslo, Norway
Ø Vatne, MD. Central Hospital, Førde, Norway.
I Bachmann, MD, Ph.D. Department of Dermatology, Haukeland University Hospital
LK Dotterud, MD, Ph.D. Hudlegekontoret Lillehammer AS, Nymosvingen 2, 2609 Lillehammer

# PROTOCOL SYNOPSIS

Compound: Methyl-aminolevulinate (MAL, Metvix®)

Study code: NTNU 2011-01

EudraCT: 2011-004797-28 NO 20120327

Study title: A randomized, controlled-blinded, multi-centre study of photodynamic therapy with methyl-aminolevulinate comparing a simplified regime with the approved regime in patients with clinical low-risk superficial and nodular basal cell carcinoma

Study centres: The study will intend to include 9 centres in Norway.

Study period:

| | |
|---|---|
| Start of recruitment | Q1 2012 |
| End of recruitment | Q3 2012 |
| Last treatment | Q4 2012 |
| End of 12-months follow-up | Q4 2013 |
| End of 36-months follow-up | Q4 2015 |

Objectives The primary objective is to compare BCC lesion response rate to a schedule using one single treatment of Metvix® PDT session with re-treatment of non-complete responders by 3 months with the response of two standard Metvix® PDT treatment sessions one week apart.

Secondary objectives are to investigate the treatment response in relation to clinical and histological tumour characteristics as tumour thickness, subtype and immunohistochemical markers.

Study design Randomized, controlled blinded multi-centre study.

Procedure Lesion mapping will take place before treatment and at the preset follow-up visits by recording the number and location of the lesions in the CRF. Registration of the lesion clinical extent, location, thickness and subtype will be done at baseline and marked on a form and/ or photographed for later identification. In order to facilitate access of the Metvix® cream and light to all parts of the lesion, the surface of BCC lesions with 5 mm of the surrounding tissue of normal appearance should be prepared using a small dermal curette. The curettage should be performed by scraping in a checked pattern for optimal removal of scales and crusts, and to roughen the surface of the lesion. Thicker lesions require debulking. Metvix® cream will then be applied in a 1.0 mm thick layer to the whole treatment field and then covered using a dressing to avoid any ambient light exposure. Metvix® is applied for 3 hours before the dressing and remnants of cream are removed. Anaesthesia using subcutaneous infiltration anaesthesia without adrenaline will be given individually at the discretion of the investigator. The treatment area is then illuminated using Aktilite CL 128

(Photocure ASA) as a light source; peak wavelength around 631 nm (redlight) and light dose 37 J/cm$^2$ and exposure time 7-9 minutes. After light treatment each lesion will be randomized to one or two possible treatment regimes; a single MAL-PDT treatment session with re-treatment if there is a non-complete response after 3 months (regimen 1), or two treatment sessions separated by one week (regimen 2).
Two independent pathologists will examine the HES stained diagnostic punch biopsies. Tumour thickness and subtype will be recorded. Various immunohistochemical stains will be used to evaluate their utility for enhanced detection of residual tumours.

Endpoints

The primary endpoint will be lesions response rate and cosmetic outcome determined by clinical assessment (visual inspection and palpation) 1 and 3 years after Metvix® PDT treatment. Clinically suspected recurrences will be examined by histology. We will report the number of lesion in need of a re-treatment 3 months following the first treatment session in regime 1. Further, report on number and time of lesion to relapse by either regime, 1 or 3 years after PDT and report on adverse events in a period of up to 3 months following last treatment session.

The secondary endpoint will be to investigate relationship between clinical and pathological tumour characteristics and tumour recurrences and histopathological tumour features as BCC tumour thickness and subtypes. Tumour thickness and subtype will be established by investigation of the prepared slides from the diagnostic punch biopsies. Using immunohistochemical techniques various antibody-targeted properties will be studied in recurrences and tumours with complete response. Several tumour related markers to examine the expression pattern of markers accordingly to the invasiveness and histopathological differentiation of BCC will be studied.

Patients

Females and males above 18 years with primary, histological confirmed BCC lesions located outside mid-face-H-area.

Sample size

Patients with at least 374 lesions (187 in each group) will be included in the study.

Statistics

Simple tabulation as well as logistic regression with lesion recurrence (yes/no, a binary variable) at 3 years will be employed to analyze the outcome. As one patient may yield more than one lesion, a mixed effects model with patient identity as random factor will be considered. Different lesions within the same patient will be subject to stratified randomization. The expected cure rate at 3 years (reference) is set to 80 %. The study aims to detect an experimental success rate of no less than 70 % (the non-inferiority margin 10 %). With a sample size of 170 lesions per group, a difference between the two treatment regime groups of at least 10% can be found with a significance level of 5% and a power of 80%.

# LIST OF ABBREVIATIONS

| | |
|---|---|
| AE | adverse event |
| BCC | basal cell carcinoma |
| CR | complete response |
| EC | ethics committee |
| EMEA | The European Agency for the Evaluation of Medicinal Products |
| GCP | Good Clinical Practice |
| ICH | International Conference of Harmonisation |
| J | Joule |
| non-CR | non-complete response |
| MedDRA | Medical Dictionary for Regulatory Activities |
| METVIX® | methyl 5-aminolevulininate hydrochloride |
| PDT | photodynamic therapy |
| PpIX | protoporphyrin IX |
| SAE | serious adverse event |
| SDV | source data verification |
| UV | ultraviolet |
| CRF | case report form |
| LED | light emitting diode |
| ITT | intend to treat |
| PP | per protocol |

All dimensional units are in standard SI units.

# TABLE OF CONTENTS


**1 INTRODUCTION .......... 4**

1.1 CLINICAL EXPERIENCE .......... 4
1.2 BENEFITS AND RISKS .......... 5
1.3 CLINICAL TRIAL REGULATIONS .......... 6

**2 OBJECTIVES OF THE TRIAL .......... 6**

2.1 PRIMARY OBJECTIVES .......... 6
2.2 SECONDARY OBJECTIVES .......... 6

**3 INVESTIGATIONAL TRIAL DESIGN .......... 6**

3.1 STUDY ENDPOINT(S) .......... 6
3.2 COMPARATIVE TREATMENT REGIMES .......... 7
3.3 RANDOMISATION AND BLINDING .......... 7
3.4 STUDY FLOW CHART .......... 7
3.5 DESCRIPTION OF INVESTIGATIONAL DRUG .......... 7
3.6 DRUG ORDERING AND STORAGE .......... 7
3.7 STUDY DURATION .......... 7
3.8 TRIAL TIMETABLE .......... 7

**4 PATIENT AND LESION SELECTION .......... 8**

4.1 NUMBER OF LESIONS .......... 8
4.2 PATIENT SCREENING .......... 8
4.3 INCLUSION CRITERIA .......... 8
4.4 PATIENT EXCLUSION CRITERIA .......... 8
4.5 PATIENT WITHDRAWAL .......... 8

**5 TREATMENT PROCEDURE .......... 9**

5.1 PRE-TREATMENT EVALUATION .......... 9
5.2 RANDOMISATION AND EMERGENCY CODE .......... 9
5.3 PRE-TREATMENT PREPARATION .......... 9
5,4 PHOTODYNAMIC THERAPY WITH METVIX® 160 MG/G CREAM AND AKTILITE (RED LIGHT) .......... 9
5.5 DRUG ACCOUNTABILITY .......... 9
5.6 FOLLOW UP EVALUATION .......... 10
*5.6.1 Efficacy follow-up* .......... 10
*5.6.2 Safety follow-up* .......... 10
5.6 PATIENT COMPLIANCE .......... 11

**6 ASSESSMENT OF EFFICACY .......... 11**

6.1 LESION RESPONSE .......... 11

**7 ASSESSMENT OF SAFETY .......... 11**

7.1 SERIOUS ADVERSE EVENT .......... 11
*7.1.1 Definition of Serious Adverse Event* .......... 11
*7.1.2 Reporting of Serious Adverse Event* .......... 12
7.2 ADVERSE EVENT .......... 13
*7.2.1 Definition of Adverse Event* .......... 13
*7.2.2 Reporting of Adverse Event* .......... 13
*7.2.3 Assessment of Adverse Event* .......... 13
*7.2.4 Severity of Adverse Event* .......... 13
*7.2.5 Relationship of Adverse Event to the new treatment regime using MAL-PDT* .......... 14
*7.2.6 Localization of Adverse Events* .......... 14
*7.2.7 Onset of Adverse Events* .......... 14

**8 STATISTICAL EVALUATION .......... 14**

8.1 STUDY DESIGN .......... 14
8.2 STATISTICAL ANALYSIS .......... 14

*8.2.1 Disposition of Lesionss* ........ *15*
*8.2.2 Efficacy* ........ *15*
*8.2.3 Safety* ........ *16*
8.3 JUSTIFICATION OF SAMPLE SIZE ........ 16

**9 DATA MANAGEMENT ........ 16**

9.1 SOURCE DATA IDENTIFICATION AND SOURCE DATA VERIFICATION ........ 16
9.2 SUBJECT DATA PROTECTION ........ 17
9.3 DATA HANDLING ........ 17

**10 ADMINISTRATIVE PROCEDURES AND ETHICAL CONSIDERATIONS ........ 17**

10.1 INSURANCE ........ 17
10.2 ETHICS COMMITTEE / INSTITUTIONAL REVIEW BOARD ........ 17
10.3 SIDE EFFECTS ........ 18
10.4 BENEFITS AND DISADANTAGES ……….. ........ 17
10.5 PATIENT INFORMED CONSENT ........ 18
10.6 REGULATORY AFFAIRS ........ 18
10.7 TRIAL MONITORING ........ 18
10.8 TRIAL AUDITS & INSPECTIONS ........ 19
10.9 FINANCING ........ 19

**11 CONFIDENTIALITY AND COMMUNICATION OF RESULTS ........ 19**

11.1 PUBLICATION ........ 19

**12 REFERENCES ........ 19**

**13 APPENDIX ........** ERROR! BOOKMARK NOT DEFINED.**20**

# 1. INTRODUCTION

## 1.1 Clinical Experience

Basal cell carcinoma (BCC) is the most common malignant skin lesions in the adult, white population (1). It is a slow-growing tumour, which despite low metastatic potential can cause significant local tissue destruction and patient morbidity(2). Nodular BCCs are typically pearly pink or flesh colored papules or nodules with telangiectasia. The superficial type occurs in varying numbers and size as reddish patches with sharp and irregular borders and showing fine scaling or crusts. Given that BCC has a predilection for sun-exposed skin on head, face and neck, cosmetic outcome is significant for choice of therapy

The most common modalities in the treatment of BCC are surgery, cryotherapy, curettage and electrodessication, radiotherapy, local chemotherapy and photodynamic therapy (PDT)(3). Metvix® cream 160 mg/g (Galderma, France) (MAL) was approved for marketing in Norway in 2003 for methyl-aminolevunlinate (MAL)-PDT and is today commercial available in more than 30 countries worldwide. Metvix® is a marketed product for treatment of actinic keratosis, BCC and Bowen’s disease.

PDT exerts the selective destruction of abnormal cells through light activation of a photosensitizer in the presence of oxygen. The photosensitizer generates reactive oxygen species upon illumination with red light that causes tissue destruction and necrosis(4).
As BCC lesions are usually located to cosmetically sensitive skin areas, PDT is an attractive treatment modality, which also allows treatment of large areas, with a high response rate and excellent cosmetic result (5, 6).

Conventional PDT of BCC lesions include 3 hours methyl aminolevulinat (MAL) cream application under occlusion, leading to high and selective accumulation of photoactive porphyrins in the BCC lesions. The porphyrins are then activated by red light illumination, thus starting a photochemical reaction which leads to cell necrosis and apoptosis. MAL-PDT for BCC is currently approved for a procedure using two treatment sessions one week apart, and is therefore considered quite time- and resource-consuming. Introducing a single treatment session, with a new PDT session for treatment failures after 3-month, might represent an attractive simplification.

Treatment with PDT has been shown to be effective for BCC with tumour clearance rates using ALA- and MAL-PDT ranging from 76 % to 100 % in superficial lesions and from 64% to 92% for nodular tumours, which are comparable to results shown with other more established treatment modalities for BCC. The best results for nodular BCCs have been obtained by repeated ALA-PDT treatment sessions, leading to 100% cure rate in 25 tumours (7). Routine double treatment sessions are today an acknowledged practice as considered to contribute to a high treatment response (8).

Results from various PDT studies are difficult to compare because the treatment procedures are not standardized and information on tumours characteristics are often lacking.
However, the experince from clinical experience and reports from several studies indicate that a significant number of BCC only require one treatment session (6).  In a randomized 5-year follow-up study a high 75% complete response rate after one session of PDT was demonstrated in superficial BCC (9).  This is promising and suggests that all tumours are not

in need of a second treatment. Introducing a single treatment session, with a new PDT session only for those tumours showing treatment failures after 3-month, might represent an attractive simplification of the current regime.

One of the challenges with topical PDT is the treatment of thick BCC lesions. The delivery of sufficient photosensitizer and light to the full depth of the lesion is critical. Improved treatment response can be achieved by careful lesion preparation(10).

Recent studies have suggested that the recurrent tumours often appear among lesions located within the central face (H-area), large lesions and among recurrent lesions themselves. Some subtypes of BCC tumour appear to have a reduced response to PDT. Morpheic BCCs, surrounded by abundant collagen fibres, generally do not respond well to topical PDT (11). Furthermore, the diminished treatment results of pigmented BCC are most likely due to inhibition of melanin molecules on the light penetration. Long-term efficacy of PDT among various BCC subtypes and tumour of various thickness is needed. The use of immunohistochemical stains may allow for an enhanced detection of tumours more resistant to therapy (12).

## 1.2 Benefits and Risks

Metvix® PDT is an approved and marked treatment modality for patients with pre-malignant disease and BCC.

General benefits of topical Metvix® PDT include:
- Non-invasive treatment available on an outpatient basis
- Several separate lesions can be treated simultaneously
- The same lesion(s) can be treated repeatedly with success
- Excellent cosmetic results
- No known toxicity or interaction with other medication
- Lesion selective, leaving the surrounding tissue intact
- No skin photosensitization after 24 hours
- Only minor, manageable local side effects related to treatment site

Study benefits include the following:
If the effect of the new, alternative and simpler treatment regime proves as effective as the standard approved regime MAL-PDT will be an even more attractive therapy alternative for low-risk BCC. The treatment will be easier to manage with improved cost-effectiveness as well as reduced discomfort and fewer side effects for the patients.

The risks attributed to Metvix® PDT are few and related mainly to transient discomfort and pain during and shortly after illumination. Inflammation following tumour destruction may give local, transient erythema and oedema. Treatment of large lesions may cause severe erythema, followed by ulceration. These side effects normally last for a few days, in rare occasions up to a week after treatment.

Standard Metvix® PDT in BCC is today given as two treatment sessions separated by one week. Patients with non-complete response after one PDT session in treatment regime 1 are offered an additional PDT at the 3-month control. The possible delay of the second session of BCC treatment is acceptable, as BCC grows very slowly

Patients with non-complete response after either PDT treatment regimes at the control visit 3 months after the latest treatment session are withdrawn from the study. They are then offered standard BCC treatment at the discretion of the dermatologist. Patients will receive detailed oral and written information about the risks of side effects. If the patients find the side effects unacceptable, they are free to withdraw from the study at any time and without any further explanation.

### 1.3 Clinical Trial Regulations

The clinical trial will be conducted in compliance with the protocol, according to ICH E6: Good Clinical Practice: Consolidated guideline, CPMP/ICH/135/95, and national guidelines including Clinical Trials Directive 2001/20/EC.

# 2. OBJECTIVES OF THE TRIAL

### 2.1 Primary Objectives

The primary objective of this study is to investigate if a more simple and flexible PDT treatment schedule (one single treatment session with re-treatment of non-complete responders-regime 1) for low-risk primary superficial or nodular BCC will be as effective as the standard and approved two-treatments one week apart (regime 2).

### 2.2 Secondary Objectives

To investigate relationship between clinical and pathological tumour characteristics and the relationship between tumour recurrence rates with tumour thickness and histopathological subtypes and various immunhistochemical markers.

# 3. INVESTIGATIONAL TRIAL DESIGN

### 3.1 Study Endpoint(s)

The primary endpoint will be lesions cure rate and cosmetic outcome by treatment regime 1 compared to lesions cure rate by treatment regime 2, assessed 36 months after PDT.
The number of lesion in need of a re-treatment 3 months following the first treatment session in regime 1 will be reported. Clinical suspected recurrences are confirmed by histology. Lesion response rate is defined as number of lesions in complete response at 12 and 36 months of follow-up. We will report on adverse advents occurring in relation to both treatment regimes from start of treatment to 3 months following last treatment. We will report on lesion recurrences among lesions treated with either regime 12 and 36 months after PDT. Cosmetic outcome will be determined by clinical assessment (visual inspection and palpation) 12 and 36 months after treatment. The results will be recorded on a 4-point ordinal scale as either excellent (absence of any stigmata other than scar formation after diagnostic punch biopsy), good (slight presence of fibrosis, atrophy or change of pigmentation), fair (moderate presence of fibrosis, atrophy or change of pigmentation) or poor (marked presence of fibrosis, atrophy or change of pigmentation).

The secondary endpoint will be to investigate relationship between clinical and pathological tumour characteristics and tumour recurrences rates, tumour thickness, histopathological BCC subtypes and immunohistochemical markers. Recurrence rate defined both as”raw” (total number of recurrences divided by the total number of tumours treated) and”strict recurrence rate” (total number of patients with recurrence divided by number of lesions observed for at least 36 month) will be reported.
Recurrence rate in relation to BCC subgroup analyses including clinical (superficial or nodular tumour), tumour size and histological characteristics will also be reported. BCC will on the basis of histological growth pattern be divided into two main types (aggressive or non-aggressive type). Tumours will additionally be studied with various immunohistochemical markers for expression of cell proliferation, invasiveness, vascularisation and growth factors.

### 3.2 Comparative treatment regimes

Two different treatment regimes of Metvix® PDT in patients with clinical superficial and nodular BCC will be compared.

### 3.3 Randomization and blinding

Lesions will be randomized to either treatment regime 1 or 2 after having signed the informed consent form and having received treatment by light. An independent, blinded dermatologist with no knowledge about the randomisation for treatment will do the evaluation of the lesions after 3 months, 1 and 3 years.

### 3.4 Study flow chart

For study flow chart, see **page 20**

### 3.5 Description of Investigational Drug

Metvix® 160 mg/g cream is supplied in 2 g collapsible tubes. The strength is given as the concentration of the active entity, methyl-aminolevulinate, which is present as the hydrochloride. The colour of the cream is cream to pale brown.

Commercial cream will be used, and the information given in the packaging will be applicable. The cream is not specially prepared for this study.
The cream will be used in the clinics only

### 3.6 Drug Ordering and Storage

The cream will be stored securely in a refrigerator at 2-8°C.
Metvix® 160 mg/g cream will be supplied free of charge to the patients.

### 3.7 Study Duration

The patients will be asked to participate in the study for 3 years. Active treatment is given one to two times during a 3-month period. The follow-up visits occur at pre-fixed times during the 3 years period after treatment. Each patient will be followed for 3 years with regards to efficacy parameters and 3 months with regard to adverse events.

### 3.8 Trial Timetable

The anticipated timetable for the trial is the following: New timetable necessary

| | |
|---|---|
| Start of recruitment | Q1 2012 |
| End of recruitment | Q3 2012 |
| Last treatment | Q4 2012 |
| End of 1 year follow-up | Q4 2013 |
| End of 3 year follow-up | Q4 2015 |

# 4.Patient and lesion selection

### 4.1 Number of lesions

277 patients with 374 lesions will be included.

### 4.2 Patient Screening

Eligible patients will be informed about the possibility to participate in this study. Before any trial related procedures are performed, the patient must be thoroughly informed about the study and he/she must sign and date the informed consent form.

### 4.2 Inclusion Criteria

Patients

- Males or females above 18 years of age.
- Written informed consent.

Lesions

- One or more primary histologically verified BCC, clinically assessed as of either superficial or nodular type.

### 4.3 Exclusion Criteria

Patients

- Woman with child-bearing potential
- Patients with Gorlin's syndrome, porphyria, xeroderma pigmentosum, history of arsenic exposure or known allergy to MAL
- Concomitant treatment with immunosuppressive medication
- Patients with physical or mental conditions that most likely will prevent them from attending the follow-up visits

Lesions

- Located within the mid-face-H-area
- Longest diameter >15 mm on face and scalp, >30 mm on the trunk and >20 mm on the limbs
- Any clinically evaluated pigmented or morpheaform lesions
- Any lesions with prior treatment

### 4.4 Patient Withdrawal

Completion or trial termination for any reason will be fully documented in the CRF page.

Patients are free to withdraw from the trial at any time without providing reason(s) for withdrawal and without prejudice to further treatment. The reason for withdrawal may be withdrawal of consent, treatment failure, adverse event(s) or loss to follow-up. The reason(s) will be recorded in the CRF.

Patients who withdraw will not be replaced.

Patients withdrawing from the trial should be encouraged to go through the same final evaluations as patients completing the trial according to the protocol with special focus on safety. The aim is to record data in the same way as for patients who complete the trial. Otherwise data will be recorded as having been consented by the patient. This will be recorded in the patient notes.

# 5. TREATMENT PROCEDURE

### 5.1 Pre-treatment Evaluation

Pre-treatment evaluation at visit 1 will only be performed after the patient has agreed to participate and has signed and dated the informed consent form. No treatment will be initiated before the signed consent has been given.

Pre-treatment evaluation will be performed according to inclusion and exclusion criteria.

### 5.2 Randomisation and Emergency Code

Lesions will be randomized to treatment regime 1 or 2 after treatment by light. In case of multiple BCC, the investigator will number the lesions. The first lesion will be randomized and the next lesion will get the other regime, implying that every second lesion is randomized. The coordinating investigator will supply the randomization code. This is an open study and no emergency code will be required.

### 5.3 Pre-treatment preparation

The treatment area will be defined and marked on a chart and/or photographed, defining each lesion relative to “landscape markers” (i.e. eyebrow, ear etc.) for easier localization at later visits.

Each lesion will be numbered and recorded in the CRF.

Preparation of the lesion surface prior to PDT is a common practice and is believed to contribute to an enhanced penetration of the cream in skin. When performing curettage using a sharp curette, superficial hard keratotic tissue is removed.

The surface of every BCC lesions with 5 mm of the surrounding tissue of normal appearance will be prepared using a small dermal curette. The curettage will be performed by scraping in a checked pattern for optimal removal of scales and crusts, and to roughen the surface of the lesion. Thicker lesions require debulking.

### 5.4 Photodynamic Therapy with Metvix® 160 mg/g cream and Aktilite® (red light)

Upon having prepared the treatment area through curettage, Metvix® 160 mg/g cream will be applied as a 1-mm thick layer on the entire treatment area. The area is covered with a plastic film and on the outside with an occlusive bandage. The cream is left for 3 hours before removed and the treatment area exposed to light using light from light-emitting diodes (Aktilite®) with a light dose of 37J/cm$^2$ and the exposure time 7-9 minutes

A corresponding treatment session is carried out after 1 week in patients randomized to treatment regime 2. For patients in treatment regime 1; an assessment of treatment response is carried out 3 months after the initial treatment session and only lesions showing non-complete response will be given a second treatment session.

### 5.5 Drug Accountability

Each investigator will be responsible for drug accountability. For each patient treated, the batch number of the tube used must be documented.

### 5.6 Follow Up Evaluation

#### *5.6.1 Efficacy follow-up*

Treatment response will be assessed at the follow-up visits; 3, 12 and 36 months after treatment. The localization of the treatment sites will be made from the body chart made and the photos taken at baseline.

#### *5.6.2 Safety follow-up*

In case of any adverse event patients should take contact with their G.P. or the local hospital for further consultation. Adverse event will be recorded in the CRF at the follow-up visits if related to treatment, according to the investigator

Follow-up:

| **Treatment regime/ lesions** | **Follow-up/ months after treatment** |
|---|---|
| Regime 1/ all lesions | 3 months after 1 treatment session |
| Regime 1/ lesions with non-complete response 3 months after first session with a re-treatment | 3 months after 2 treatment session |
| Regime 1/ all lesions in complete response | 12 months after 1 or 2 treatment sessions |
| Regime 1/ all lesions in complete response | 36 months after 1 or 2 treatment sessions |
| Regime 2/ all lesions | 3 months after 2 treatment session |
| Regime 2/ all lesions in complete response | 12 months after 2 treatment sessions |
| Regime 2/ all lesions in complete response | 36 months after 2 treatment sessions |

### 5.7 Patient Compliance

Healthcare professionals in the clinic perform the entire treatment. The cream will be applied to the entire treatment area and 5 mm of the surrounding tissue of normal appearance Within 3 hours after cream application; the lesion will be exposed to red light using a lamp with light-emitting diodes (Actilite®) for 7-9 minutes.

# 6. ASSESSMENT OF EFFICACY

### 6.1 Lesion Response

The primary objective of this study is to record BCC lesions response rate to two different treatment regimes using MAL-PDT.

Lesions response rate is defined as number of lesions in clinical complete response at follow-up. Follow-up will occur 3, 12 and 36 months after treatment and is fully described under section 5.6.2. In the case of lesions of clinical non-complete response, a histological investigation will be carried out. Patients with histologically remaining BCC will be excluded from the study.
The investigators will be asked to assess whether each lesion response fits into one of the following two categories:

| | |
|---|---|
| **Complete response (CR):** | Complete disappearance of the lesion, visually and by palpation (mild erythema /pigmentation might remain) |
| **Non-complete response (Non-CR):** | Remaining lesion |

# 7. ASSESSMENT OF SAFETY

### 7.1 Serious Adverse Event

#### *7.1.1 Definition of Serious Adverse Event*

A serious adverse event (experience) or reaction is any untoward medical occurrence that at any dose:

1) results in death
2) is life threatening

   NOTE: The term “life-threatening” in the definition of “serious” refers to an event in which the patient was at risk of death at the time of the event; it does not refer to an event, which hypothetically might have caused death if it were more severe.

3) requires inpatient hospitalization or prolongation of existing hospitalization[1]

[1] Complications occurring during hospitalisation are AEs and are SAEs if they cause prolongation of the current hospitalisation. Hospitalisation for elective treatment of a pre-existing non worsening condition is not, however,

4) results in persistent or significant disability/incapacity[2], or
5) is a congenital anomaly/birth defect

In addition, medical and scientific judgment is required to decide if prompt notification is required in other situations, i.e. any event which the investigator regards as serious that did not strictly meet the criteria above but may have jeopardized the subject or required intervention to prevent one of the outcomes listed above, or which would suggest any significant hazard, contraindication, side effect or precaution that may be associated with the use of the drug.

### *7.1.2 Reporting of Serious Adverse Event*

The investigator must report any SAE occurring between the treatment with study drug and completion of 3-month follow-up after last treatment, whether or not considered related to study drug to the sponsor.

Pregnancies occurring during the study, although not SAEs, should be reported using the SAE reporting procedures.

The SAE should be reported by telephone and/or fax **within 24 hours** following knowledge of the event by the investigator for the attention of **Eidi Christensen:**

Telephone: 72 82 20 50
Fax: 72 82 20 74

The investigator should not wait to receive additional information to fully document the event before notifying a SAE, although additional information may be requested. Where applicable, information from relevant laboratory results, hospital records and autopsy reports should be obtained. The investigator is also required to submit follow-up reports until such time as the AE has resolved or in the case of permanent impairment, until the AE stabilizes.

Instances of death, congenital abnormality or an event that is of such clinical concern as to influence the overall assessment of safety, if brought to the attention of the Investigator at any time after cessation of study medication and linked by the Investigator to this study, should be reported.

The sponsor will report the SAE to the relevant authority within the required timeframe, depending on the local regulations, electronically where possible. Sponsor will report all serious and unexpected adverse drug reactions in an expedited fashion to all concerned investigators. Sponsor together with the investigators are responsible for reporting serious and unexpected adverse drug reactions to the Independent Ethics Committees (IECs) or Institutional Review Board (IRB), where required.

Details of SAEs will also be reported on the adverse event pages in the CRF.

---

considered an AE. The details of such hospitalisations must be recorded on the medical history/physical examination page of the CRF.

[2] An AE is incapacitating or disabling if it results in a substantial and/or permanent disruption of the patient's ability to carry out normal life functions.

## 7.2 Adverse Event

### *7.2.1 Definition of Adverse Event*

An adverse event (AE) is any untoward medical occurrence in a patient or clinical investigation subject administered a pharmaceutical product and which does not necessarily have a causal relationship with this treatment.

An adverse event can therefore be any unfavourable and unintended sign (including an abnormal laboratory finding), symptom, or disease temporally associated with the use of a medicinal (investigational) product, whether or not related to the medicinal (investigational) product.

All events occurring after the subject has signed the study consent form but before receiving the drug/procedure will be registered as clinical symptoms at baseline.

### *7.2.2 Reporting of Adverse Event*

Local phototoxic reactions like pain, erythema and pustular eruption, and any other adverse events related to the treatment according to the investigator will be recorded in the CRF.

All reported AE's will be followed up until resolved or as clinically required.

### *7.2.3 Assessment of Adverse Event*

Adverse events may be reported spontaneously by the subject or elicited through open (non-leading) questioning during the study. As far as possible, all AE's must be described by their duration (start and stop date), severity (mild, moderate, or severe; see Section 0), relationship to treatment (yes, uncertain, no; see Section 0), and according to the need of other specific therapy.

Localization of AE's will be recorded as "treatment area" or "non-treatment area"(see Section 0). The onset of AE's will be classified relative to the stage of treatment as described in Section 0.

### *7.2.4 Severity of Adverse Event*

Adverse events will be graded as:

Mild: The AE is transient and easily tolerated.

Moderate: The AE causes the subject discomfort and interrupts the subject's usual activities.

Severe: The AE causes considerable interference with the subject's usual activities and may be incapacitating or life threatening.

### *7.2.5 Relationship of Adverse Event to new treatment regime using MAL-PDT*

The investigator's opinion of the relationship of the AE(s) to the investigational drug, will be categorized as "yes," "uncertain," or "no."

No: An AE, which after careful examination at the time of evaluation, is judged to be clearly and incontrovertibly due to extraneous causes (disease, environment, etc) and which does not meet the criteria for drug relationship and/or device relationship listed under uncertain or yes.

Uncertain: An AE, for which after careful examination at the time of evaluation, the connection with the test drug administration or illumination appears unlikely, but cannot be ruled out with certainty.

Yes: An AE, for which after careful examination at the time of evaluation, the connection to the test drug administration or illumination appears, with a high degree of certainty, to be related to the test drug and/or device.

### *7.2.6 Localization of Adverse Events*

The investigator should report localization for all AE. For any local phototoxic reaction it must be reported if the adverse event occurred in the treatment area or not.

### *7.2.7 Onset of Adverse Events*

The investigator will classify the onset of each AE relative to the following stages of treatment:

- After cream application and before light exposure
- During or immediately after light exposure
- After treatment

# 8. STATISTICAL EVALUATION

## 8.1 Study Design

Females and males above 18 years with primary and histologically verified BCC located outside of the mid-face (H-region) area with lesions of longest diameter of ≤ than 15 mm when located in the face and on the scalp, ≤ than 30 mm on the trunk and ≤ than 20 mm on the extremities, will be included. This multi-centre study will be an open label, controlled blinded, randomized and parallel-group study where the lesions are randomized to either treatment by regime 1 or regime 2. Evaluation of efficacy and evaluation of cosmetic outcome will be assessed at the follow up visits; 3 12 and 36 months after last treatment. Adverse events will be noted at the 3-month follow-up after last treatment.

## 8.2 Statistical Analysis

The statistical analysis is the responsibility of Eirik Skogvoll, Unit for applied clinical research, Faculty of Medicine, Norwegian University of Science and Technology (NTNU),

Trondheim, Norway. Tabulation of summary statistics and data analyses will be performed using SPSS® software (PASW for Windows version 17.0 (PASW Inc)). The primary presentation and analysis will be based on data pooled across centres. Relevant summaries of data for individual centres, or combination of centres, for primary and secondary data (ie, patient characteristics, efficacy endpoints, and safety endpoints) will be made. Continuous data will be presented by descriptive statistics with the number of observations (n), mean, standard deviation, minimum, median, and maximum. Categorical data will be summarized in frequency tables using count and percentages. All tests will be carried out at a significance level of 5% without correction for multiple testing. All patients will be presented in separate data listings. Data from patients screened, but not included in the study, will not be presented in any tables or listings.

### *8.2.1 Disposition of Lesions*

The number of lesions which are included into the study, which receive Metvix® cream, which are included in safety and/or efficacy evaluation, and which complete each phase of the study, as well as withdrawals will be summarized using counts and percentages of lesions.

Each lesion will be identified, randomized, treated and followed up individually. Patient identification will be recorded anonymously, to account for potential individual susceptibility with respect to response.

The primary outcome is defined as “cure” or “no cure” of a lesion at 3 years, i.e. a binary variable. From previous experience it is expected that the reference treatment leads to a probability of cure of 80 %.

**Per-protocol (PP) efficacy population**

This population will consist of all treated BCC-lesions, except lesions considered not evaluable due to major deviations from the protocol. Major deviations may include: inclusion criteria not respected, non-available efficacy assessment, visit window discrepancy, or discrepancies from the treatment schedule. Major deviations will be assessed before start of the statistical analyses. Only observed data will be part of the per-protocol analysis. A lesion with missing response at 3-months will be excluded from the calculation of the patient response rate.

**Intent-to-treat (ITT) efficacy population:**

This population will consist of the entire population for whom any aspect of treatment with Metvix® cream was initiated. This population will be analyzed using the last observation carried forward (LOCF) method to impute missing values and to avoid possible bias introduced by non-random dropout of patients. A lesion with missing response at 3-months, will be handled as non-complete response in the calculation of the patient response rate.

### *8.2.2 Efficacy*

The primary endpoint will be lesion response rate determined by clinical assessment (visual inspection and palpation) 3, 12 and 36 months after Metvix® PDT treatment. Lesion response rate is defined as number of lesions in complete response at the follow-up.

The primary hypothesis to be tested will be:

$$H_0: \mu_1 \leq \mu_2 - \delta_0 \text{ vs. } H_A: \mu_1 > \mu_2 - \delta_0$$

Here $\mu_1$ = expected response rate for treatment regimen 1, $\mu_2$ = expected response rate for treatment regimen 2, $\delta_0$ = 0.1, non-inferiority margin.

As one patient may yield more than one lesion, a mixed effects logistic regression model with patient identity as random factor will be considered. Different lesions within the same patient will be subject to stratified randomization.

A two-sided 95% confidence interval for difference in lesion response rate will be calculated and supplemented by odds ratio from the logistic regression. The calculation of the confidence interval will be based on data pooled across centers.

Efficacy results will be presented for the ITT and for the PP population.

#### *8.2.3 Safety*

The reported adverse events (including local reactions) will be coded according to MedDRA terminology. The number and percentages of subjects with at least one adverse event, and the relationship will be tabulated. Occurrence of particular adverse events (by body system and preferred MedDRA term) and their severity and relationship will be summarized using counts and percentages of subjects.

The erythema and pustular eruption scores with 4 categories will be summarized by treatment regime group and time point using count and percent.

### 8.3 Justification of Sample Size

The cure rate at 3 years with conventional treatment (5, 6) is expected to be 85 %. The study aims to detect an experimental success rate that is less than 75 % (i.e. a non-inferiority margin of 10 %). With a significance level of 5% and a power of 80% a sample size of 170 lesions per group is required (ref StatExact 8, Cytel Corp. USA). To accommodate some loss to follow-up, we aim to recruit a total of 187 lesions in each group.

# 9. DATA MANAGEMENT

### 9.1 Source Data Identification and Source Data Verification

Patient information collected in the CRF, but not recorded in the patient notes, is regarded as source data. However, the patient's participation and any serious adverse events related to the study treatment should be documented in the patient hospital files.

In the process of ensuring data completeness and accuracy, source data verification (SDV) should be performed. The patients will be informed in writing about the need for source data verification (SDV). SDV will be performed by a monitor appointed by the coordinating investigator. To be able to do SDV, monitor will require and review relevant part of the patient hospital files.

### 9.2 Subject Data Protection

Patient number, initials, date of birth and sex will identify the patients in the CRFs.

The investigator is responsible for keeping a list of all randomized patients including patient numbers, full names and date of birth. In addition, the investigator will prepare a list of patients who were screened for participation of the trial but were not randomized and the reason for non-eligibility.

The patients will be informed in writing that the results will be stored and analyzed in a computer according to national laws, as applicable, and that patient confidentiality will be maintained.

### 9.3 Data Handling

The investigator or his/her designee will, on the individual CRF document all data obtained during the study.

The reasons for any missing data must be noted on the CRF. Corrections should be made legibly, dated and initialled. Incorrect entries must not be covered by correction fluid, or obliterated, or made illegible in any way.

For all patients the monitor appointed by the coordinating investigator will collect the completed CRFs. Each site will retain a copy of the signed CRF. If the monitor detects CRFs with missing or inconsistent data not catered for, queries will be sent to the site for correction. Each investigator will also receive copies of any data query forms, which might be generated during the data validation process.

Source data, source documents, CRF, protocol and amendments, drug accountability forms, correspondence, patient identification list, informed consent forms, and other essential GCP documents must be retained for at least 15 years after the study is completed.

Patient data will be entered continuously into the database by personal appointed by the coordinating investigator.

All original documents and copies of the CRF will be retained at each site.

## 10. ADMINISTRATIVE PROCEDURES AND ETHICAL CONSIDERATIONS

### 10.1 Insurance

All patients in the study will be coved by insurance as a separate insurance for the study.

### 10.2 Ethics Committee / Institutional Review Board

The trial protocol, including the subject information and informed consent to be used, must be approved by the regional EC. Written approval must be obtained before enrolment of any subjects into the trial.

The principal investigator will ensure that this study is conducted in full conformance with the Edinburgh, Scotland, (2000) amendment to the Declaration of Helsinki 1964 and with national laws and regulations for clinical research.

The investigator is responsible for informing the ethics committees and regulatory authorities of any SAE and/or major amendments to the protocol as per national requirements. The investigator should file all correspondence and notify the ethics committees and regulatory authorities when the study is completed.

### 10.3 Side effects

PDT with to sessions of Metvix® is an approved, marketed treatment of patients with BCC. PDT might be associated with local discomfort or pain during illumination and to a lesser degree after treatment. Inflammation and erythema can also be seen in the treatment area after PDT and when treating skin with severe lesions severe erythema and crusting will be expected. These side effects will disappear during a few days and in rare cases up to one week after treatment. Systemic adverse events are not suspected.

### 10.4 Benefits and disadvantages

PDT treatment is given on a out patents' clinic, hence, the travel time to treatment will for patient in need of a second treatment after 3 months be the same as if they received to sessions of treatment one week apart. Following the procedures outlined in this study a number of patients may reduce treatment to one session of PDT. A possible delay of a second treatment session of 3 months does not pose a risk of aggravated morbidity or metastatic disease in this type of skin cancer. A patient who enters the study may benefit from a close follow-up. Although the study results will be of limited benefit to the participating patients, they may contribute to a more effective treatment regime in the future.

### 10.4 Patient Informed Consent

The investigator is responsible for giving the subject and complete verbal and written information about the nature, purpose, and possible risks and benefits of the trial. Trial subjects must also be notified that they are free to withdraw from the trial at any time. The subjects should have reasonable time to read and understand the information before signing. The investigator is responsible for obtaining signed or oral EC-approved informed consent from all subjects and/or guardians before performing any trial-related procedures.

A copy of the subject information and of the subject informed consent form will be given to the subjects. The signed consent form will be kept by the investigator, either in the patient hospital file or in the investigator's study file.

Participating patients will be informed about the result of the study if they express a wish of this.

### 10.5 Regulatory Affairs

A notification will be submitted to national authorities before commencement of the trial, as applicable according to local regulations. Notifications and reports will be filed according to ICH E6(R1): GCP: Consolidated guideline, CPMP/ICH/135/95, and national guidelines including Clinical Trials Directive 2001/20/EC.

### 10.6 Trial Monitoring

Prior to the start of the study, the coordination investigator will ensure that the investigators and their staff are familiar with the protocol, CRFs and other study documents and procedures. The investigators will be visited on a regular basis by the monitor (appointed by the coordinating investigator), who will check trial procedures, including safety assessments, drug handling, data recording and SDV. The monitor will be allowed to review relevant hospital records to confirm that required protocol procedures are being followed and check consistency between patient record and CRF. Incorrect or missing entries onto the CRFs will be addressed as data queries and must be corrected immediately. Trial monitoring will not jeopardize patient confidentiality.

### 10.7 Trial Audits & Inspections

The patients will be informed in writing about the possibility for audits and/or inspections. The audit and/or inspection might be performed by at least one of the following parties hospital institutional review boards (IRB)/ethics committees or regulatory authority. In these cases relevant part of the patient records will be required and reviewed.

### 10.8 Financing

The study is not sponsored.

## 11. CONFIDENTIALITY AND COMMUNICATION OF RESULTS

### 11.1 Publication

The statistical/research report may form the basis for several manuscripts intended for publication in scientific journals. Before this agreed time, no data from the trial will be published. Preliminary data may be presented at professional medical conferences. The investigators will be given 14 days to review and comment on any manuscript/abstract or other means intended for publication or presentation of the data.

The published international guidelines for authorship (International Committee of Medical Journal Editors, 1997) will be adhered to; i.e.

> ‘All persons designed as authors should qualify for authorship. Each author should have participated sufficiently in the work to take public responsibility for the content’.

It is the intention that the coordinating investigator will be first author, and to have one investigator form each site as author, however the final decision on the order of authorship will be decided when the study has been finalized.

## 12. REFERENCES


1. Miller DL, Weinstock MA. Nonmelanoma skin cancer in the United States: incidence. J Am Acad Dermatol. 1994; 30(5 Pt 1): 774-8.

2. Crowson AN. Basal cell carcinoma: biology, morphology and clinical implications. Mod Pathol. 2006; 19 Suppl 2: S127-47.
3. Telfer NR, Colver GB, Morton CA. Guidelines for the management of basal cell carcinoma. Br J Dermatol. 2008; 159(1): 35-48.
4. Peng Q, Warloe T, Berg K, Moan J, Kongshaug M, Giercksky KE, et al. 5-Aminolevulinic acid-based photodynamic therapy. Clinical research and future challenges. Cancer. 1997; 79(12): 2282-308.
5. Morton CA, McKenna KE, Rhodes LE. Guidelines for topical photodynamic therapy: update. Br J Dermatol. 2008; 159(6): 1245-66.
6. Braathen LR, Szeimies RM, Basset-Seguin N, Bissonnette R, Foley P, Pariser D, et al. Guidelines on the use of photodynamic therapy for nonmelanoma skin cancer: an international consensus. International Society for Photodynamic Therapy in Dermatology, 2005. J Am Acad Dermatol. 2007; 56(1): 125-43.
7. Svanberg K, Andersson T, Killander D, Wang I, Stenram U, Andersson-Engels S, et al. Photodynamic therapy of non-melanoma malignant tumours of the skin using topical delta-amino levulinic acid sensitization and laser irradiation. Br J Dermatol. 1994; 130(6): 743-51.
8. Haller JC, Cairnduff F, Slack G, Schofield J, Whitehurst C, Tunstall R, et al. Routine double treatments of superficial basal cell carcinomas using aminolaevulinic acid-based photodynamic therapy. Br J Dermatol. 2000; 143(6): 1270-5.
9. Basset-Seguin N, Ibbotson SH, Emtestam L, Tarstedt M, Morton C, Maroti M, et al. Topical methyl aminolaevulinate photodynamic therapy versus cryotherapy for superficial basal cell carcinoma: a 5 year randomized trial. Eur J Dermatol. 2008; 18(5): 547-53.
10. Christensen E, Warloe T, Kroon S, Funk J, Helsing P, Soler AM, et al. Guidelines for practical use of MAL-PDT in non-melanoma skin cancer. J Eur Acad Dermatol Venereol. 2009.
11. Randle HW. Basal cell carcinoma. Identification and treatment of the high-risk patient. Dermatol Surg. 1996; 22(3): 255-61.
12. Barrett TL, Smith KJ, Hodge JJ, Butler R, Hall FW, Skelton HG. Immunohistochemical nuclear staining for p53, PCNA, and Ki-67 in different histologic variants of basal cell carcinoma. J Am Acad Dermatol. 1997; 37(3 Pt 1): 430-7.


# 13. APPENDIX

## Table 1 Study Flow Chart

| Visit number | | 1<br>Start of therapy | 1b<br>2. PDT | 2<br>3.m follow-up/2.therapy | 2b<br>3-m follow-up for extra PDT | 3<br>1-y follow-up | 4<br>3-y follow-up |
|---|---|---|---|---|---|---|---|
| Week | I | 1 | 2 | 14 | | 52 | 156 |
| | II | 1 | | 12 | | 52 | 156 |
| | III | 1 | | 12 | 24 | 76 | 180 |
| Informed consent | | X | | | | | |
| Inclusion/exclusion criteria | | X | | | | | |
| Demographic and baseline data | | X | | | | | |
| PDT | I | X | X | | | | |
| | II | X | | | | | |

| | | | | | | | |
|---|---|---|---|---|---|---|---|
| | III | X | | X | | | |
| Clinical assessment | | | | X | X[III] | X | X |
| AE | | | | X | X[III] | | |
| Cosmesis | | | | X | X[III] | X | X |
| Biopsy | | | | X* | X*[III] | X* | X* |

I: Approved PDT regime with two PDT treatments one week apart
II: New PDT regime with one PDT treatment
III: New PDT regime with one PDT treatment pluss one extra PDT treatment due to non-complete response after 3- month assessment.
*Clinically suspected recurrences will be examined by histology. Patients with histologically remaining BCC will be excluded from the study.
