# Supplementary material for: New, simplified versus standard photodynamic therapy (PDT) regimen for superficial and nodular basal cell carcinoma (BCC): A single-blind, non-inferiority, randomised controlled multicentre study
Source: PLoS One. 2024 Mar 8;19(3):e0299718. doi: 10.1371/journal.pone.0299718 (PMC10923430; doi:10.1371/journal.pone.0299718)
Supplement: Attachment — Submitted filename: Response to editor and reviewer.docx [file pone.0299718.s006.docx]

**Response to editor and reviewer**

We thank you for reviewing our paper and greatly appreciate the effort and comments provided.

The original manuscript has been revised according to the response to the comments.

Our answers to can be found under the comments and in italic.

**Comments from reviewer #3**:

Page 3 line 43: "...with objective to.." better to correct as "..with the objective of investigating.."
Table 4: per protocol analysis: the difference need to be corrected as 9.3%

*Answer:*

*The wording on page 3, line 43 has been changed to “…with the objective of investigating"*

*The number in Table 4 has been corrected from 9.4 % to 9.3%.*
